# The "Brain Stethoscope": A Non-Invasive Method for Detecting Elevated Intracranial Pressure

**DOI:** 10.7759/cureus.13865

**Published:** 2021-03-13

**Authors:** Nathan Kostick, Kim Manwaring, Rajkumar Dhar, Richard Sandler, Hansen Mansy

**Affiliations:** 1 Medicine, University of Central Florida College of Medicine, Orlando, USA; 2 Pediatric Neurosurgery, Orlando Regional Medical Center, Orlando, USA; 3 Mechanical and Aerospace Engineering, University of Central Florida, Orlando, USA; 4 Pediatric Gastroenterology, Nemours Children's Hospital, Orlando, USA

**Keywords:** non-invasive, icp monitoring, intracranial hypertension, hydrocephalus, icp

## Abstract

Introduction

Minimally invasive intracranial pressure (ICP) screening has long been desired by neurosurgeons. A novel approach deriving ICP from tympanic membrane (TM) pulsation may offer the solution. The ICP waveform appears to be transmitted to the TM by the cochlear aqueduct. The resulting TM infrasonic pulsations can be measured by certain sensors. Elevated ICP alters brain compliance, which appears to yield slower rise times of the TM pulsation waveform. Measurement of this change may be useful in screening for elevated ICP. This paper investigates one such technique.

Methods

A stethoscope was modified for airtight external ear canal fit; the dome was exchanged for a magnetic reluctance pressure sensor, allowing measurement of TM pulsations. Analog TM pulsations were analyzed by measuring the pulsation's slope ratio between the waveform’s downslope and upslope. Seventeen normal subjects (ages 18-32 years) underwent hyperventilation and tilt table testing to induce ICP changes. An algorithm processed this data and predicted the subject's ICP status.

Results

The slope ratio method showed consistent and stable changes with the expected alterations in ICP from the tilt test and hyperventilation maneuvers. The classification algorithm correctly identified subjects with elevated ICP in 60 of 60 independent recordings on 17 subjects.

Conclusion

This paper has four conclusions. First, the "brain stethoscope" can detect increased ICP from the TM pulsation waveform in healthy subjects. Second, analysis of the TM waveform using slope ratio calculations is capable of distinguishing normal versus elevated ICP. Third, the tilt and hyperventilation maneuvers showed the expected physiologic trends. Last, further studies are needed on patients with pathological ICP before the brain stethoscope can be implemented into clinical practice.

## Introduction

Elevated intracranial pressure (ICP) is commonly screened for in multiple conditions including hydrocephalus, pseudotumor cerebri, and trauma [1]. The standard of practice for measuring ICP involves lumbar puncture and measuring the cerebrospinal fluid opening pressure via manometry or through a direct intracranial interface with an external ventricular drain saline column transduced by a strain gauge [2]. This is obviously invasive and often uncomfortable for patients. Patients that require routine ICP monitoring must regularly endure this procedure [3]. There is a clear need for a minimally or non-invasive technique to screen for elevated ICP [4]. Many studies have attempted to develop non-invasive methods to identify elevated ICP using techniques such as trans-ocular ultrasound, carotid artery doppler, and cochlear aqueduct transmission [2,5,6]. However, to date, none have proven to be reliable enough for clinical practice [2,4-7]. One technique of interest is the use of tympanic membrane pulsation for ICP derivation [8,9]. The technique, first described in the 1970s, makes use of a known channel between the cerebrospinal fluid (CSF) and the middle ear via the cochlear aqueduct [10]. Many studies have shown this connection allows for the cardiac pulsation waveform (ICP waveform) to be transmitted to the tympanic membrane (TM) and allows for the ICP waveform to be derived from the TM pulsation [10-14]. Although previous tests have been able to derive this waveform, the variable acoustic properties of the cochlear aqueduct tend to render classical ICP waveform metrics, such as amplitude and time-averaged mean, unreliable [2,15]. This limitation, when combined with the bulky and complex equipment originally needed to detect these waveforms, made this technique impractical [2]. This paper describes a simple novel approach that overcomes previous limitations by utilizing new waveform metrics and modern sensors to allow for reliable detection of elevated ICP. The proposed novel waveform metrics utilize the phenomenon that abnormal intracranial compliance caused by elevated ICP leads to accelerated wave transmission through the brain resulting in a more delayed rise time in the TM pulsation [2,14]. By quantifying this rise time change via slope ratio (described in the methods section) ICP alterations can be monitored without reliance on classical waveform metrics. A device consisting of a modified stethoscope which the patient wears connected to an ultrasensitive magnetic reluctance pressure sensor was able to detect these changes in the TM pulsation. 

We evaluated the efficacy of this technique by studying its ability to identify induced alterations in ICP status on healthy subjects. Healthy subjects were chosen over subjects with pathologically elevated ICP as experimentation on subjects with pathologically changes in ICP is fraught with practical and ethical concerns. To avoid these concerns we used physiological maneuvers that have been well established to cause alterations in ICP such as tilt table testing or hyperventilation [16,17]. For example, a 45-degree tilt with a subject's head down increases ICP by 15-20 mmHg [16]. Alternatively, hyperventilation induces hypocapnia (lowered CO2) which decreases cerebral blood flow, cerebral perfusion pressure, and ICP [18]. This effect has been well established in both experimental and clinical environments and is known to cause decreases in ICP by 5-20 mmHg [17]. 

This study set out to answer the five questions. First, can the brain stethoscope detect tympanic membrane pulsation (TMP) waveforms? Second, can alterations in TMP caused by elevated ICP be detected by the brain stethoscope? Third, do we see the expected changes in TMP/SR with tilt-testing and hyperventilation? Fourth, can the slope ratio method be used to determine ICP status? Finally, does the brain stethoscope have the potential as a non-invasive technique for identifying elevated ICP?

## Materials and methods

Technical specifications

**Figure 1:**
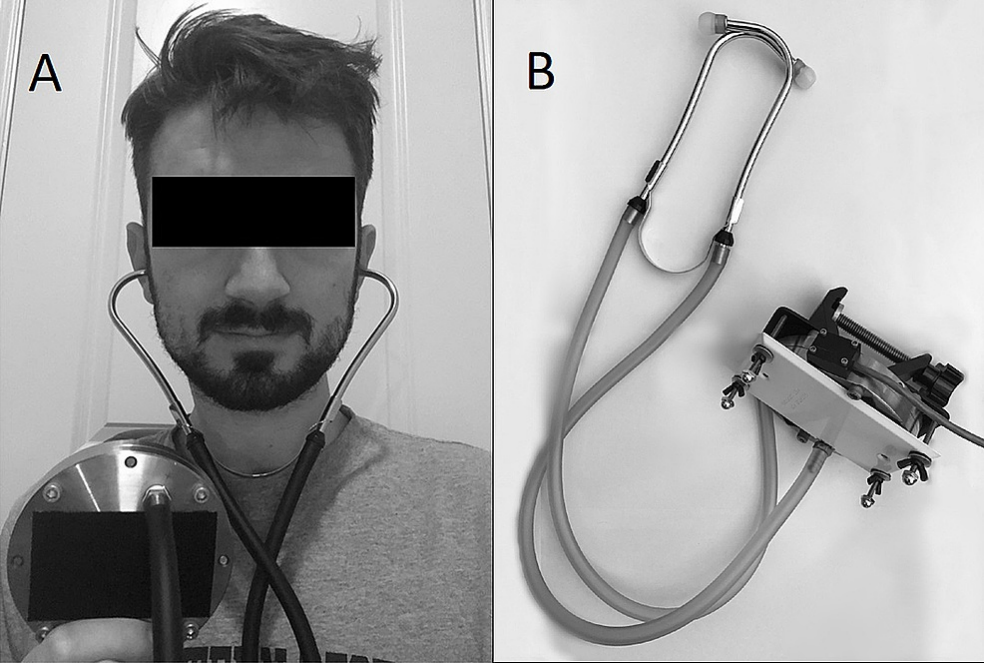
Image of the brain stethoscope (A) Subject (or patient) wearing the brain stethoscope. Tympanic membrane pulsation is transmitted from the subject's ear to the pressure transducer via the stethoscope. (B) The "brain stethoscope." The stethoscope is placed into the subject's ears, the signal is transmitted to a pressure sensor and then to a computer readout.

A Sprague Rappaport Stethoscope (ESR-112, Elite Medical Instrument Inc., Fullerton, CA) stethoscope is modified to create a sealed air column between the eardrum of the subject and a variable reluctance pressure transducers (DP103, diaphragm range dash number: 10, Validyne Engineering, Los Angeles, CA) (Figure 1). The transducer voltage output, representing the tympanic membrane pulsations, is then converted by an analog to digital converter (ADC) and fed into the real-time analysis software. A magnetic reluctance pressure sensor operates by using a flexible metal film between two coils. The metal film deforms with small changes in pressure leading to an equivalent change in deflection of the metal film and a corresponding change in reluctance and output voltage of the device. This allows high fidelity for monitoring of very small pressure changes.

Experimental methods

To establish that the brain stethoscope can detect TMP waveform, a standard stethoscope with an airtight external ear canal fit was modified by exchanged the dome for an ultrasensitive pressure sensor allowing detection of the TMP. Seventeen healthy subjects aged 18-32 years participated in the study. TMP waveform was recorded at rest for two minutes. This waveform was compared against historical TMP waveforms/ICP waveform to determine that the brain stethoscope is capable of detecting TMP.

**Figure 2:**
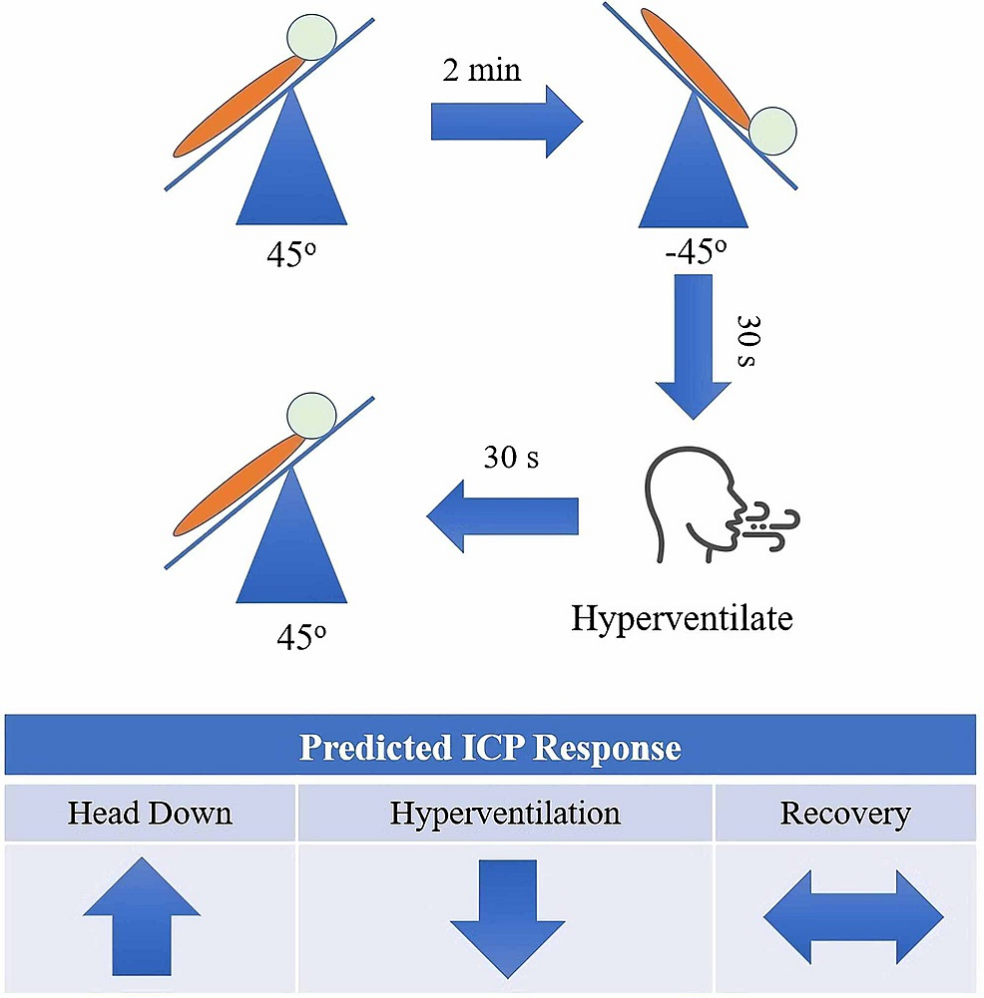
Demonstration of experimental workflow A baseline recording is taken upright. Then the subject is inverted head down at 45 degrees. The subject is then instructed to hyperventilate. Afterward, the subject is returned upright. Below is the predicted ICP response, for each provocative maneuver. ICP: intracranial pressure

To determine if alterations in ICP can be detected by the brain stethoscope, further recordings were performed after the baseline. Each subject was inverted (head down) at 45 degrees for 30 seconds. This maneuver provides a reference point for elevated ICP as tilt-testing at 45 degrees is known to elevate a subject's ICP by 15-20 mmHg [16]. The subjects were then instructed to hyperventilate to 20 mmHg CO2 per capnometry. This maneuver allows us to confirm that the TMP waveform is ICP drove and not blood pressure, as hyperventilation is known to reduce ICP by 5-20 mmHg but does not cause significant changes in blood pressure [19,20]. This means that if the waveform shows reversibility with hyperventilation it is ICP driven, but if it does not it is driven by blood pressure. Subjects were returned upright, and a two-minute post-maneuver baseline was recorded to provide a post-experimental control. TMP was continuously monitored during these maneuvers. A diagram of the complete experimental workflow can be seen in Figure 2.

To determine if the expected trends were seen with each physiological maneuver the slope ratio (SR) of a given recording was found by dividing the downslope of a waveform by its upslope and averaging this for all waveforms over a 10 second period. The slope ratio was calculated at each stage of the experiment (resting, tilt-test, hyperventilation, and post-experiment resting). A paired t-test was used to compare the SR before and after each maneuver.

To determine if the slope ratio method can be used to determine ICP status, an algorithm was developed to predict ICP status from SR. To develop this algorithm, the data from the first three subjects was used to identify an SR threshold for elevated ICP. These subjects underwent two rounds of the tilt-table testing and hyperventilation challenges described in Figure 2 with a rest period of five minutes between each recording (18 total recordings; 3 subjects x 2 rounds x 3 states). The data from this initial cohort was used to train an algorithm to classify recordings as either normal or raised ICP. The algorithm was designed to identify an SR threshold that was capable of predicting a subject’s ICP status. The next cohort of subjects only underwent one round of tilt-table testing. This algorithm was then fed the novel data (i.e., data not previously seen by the algorithm) consisting of 42 samples from 14 other subjects.

If the results of study objectives one to four were favorable, then we could conclude that the brain stethoscope has the potential as a non-invasive technique to identify elevated ICP.

This study was approved by the University of Central Florida IRB #BIO-16-12579.

## Results

**Figure 3:**
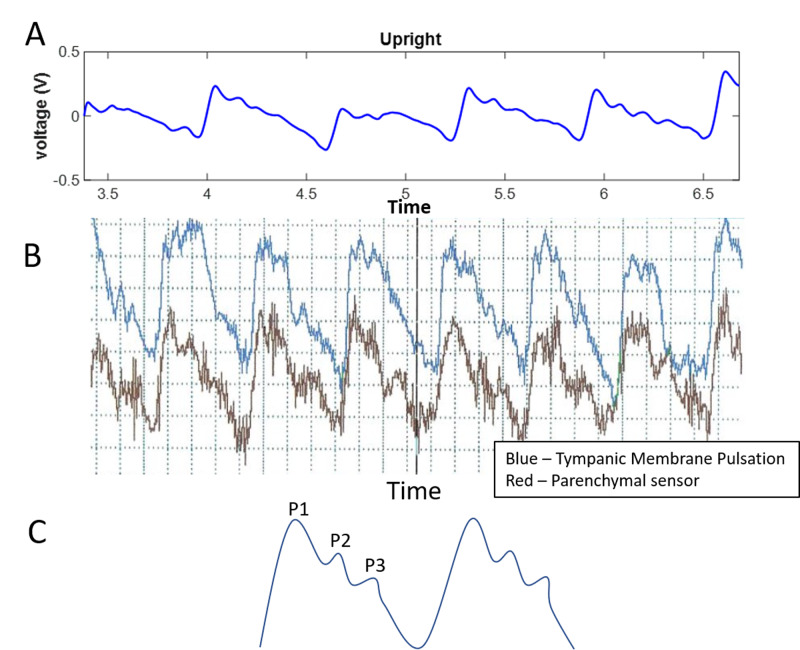
Example TMP and ICP waveforms (A) Sample TMP recording of a subject at rest. (B) Example of the previous generation TMP recording using a microphonic sensor. (C) Schematic of prototypical ICP pulse waveform. TMP: tympanic membrane pulsation; ICP: intracranial pressure

First, “can the brain stethoscope be used to detect TMP?” The TMP waveforms were able to be detected at rest in 17 of 17 subjects, an example of one of these waveforms can be seen in Figure 3A. These waveforms appear consistent in morphology to previous recordings of TMP waveforms Figures 3A, 3B. Similarly, as found in previous studies, the TMP waveform is similar in morphology to that of the ICP waveform Figures 3A, 3C showing the classic P1, P2, and P3 waves [4,8,10].

**Figure 4:**
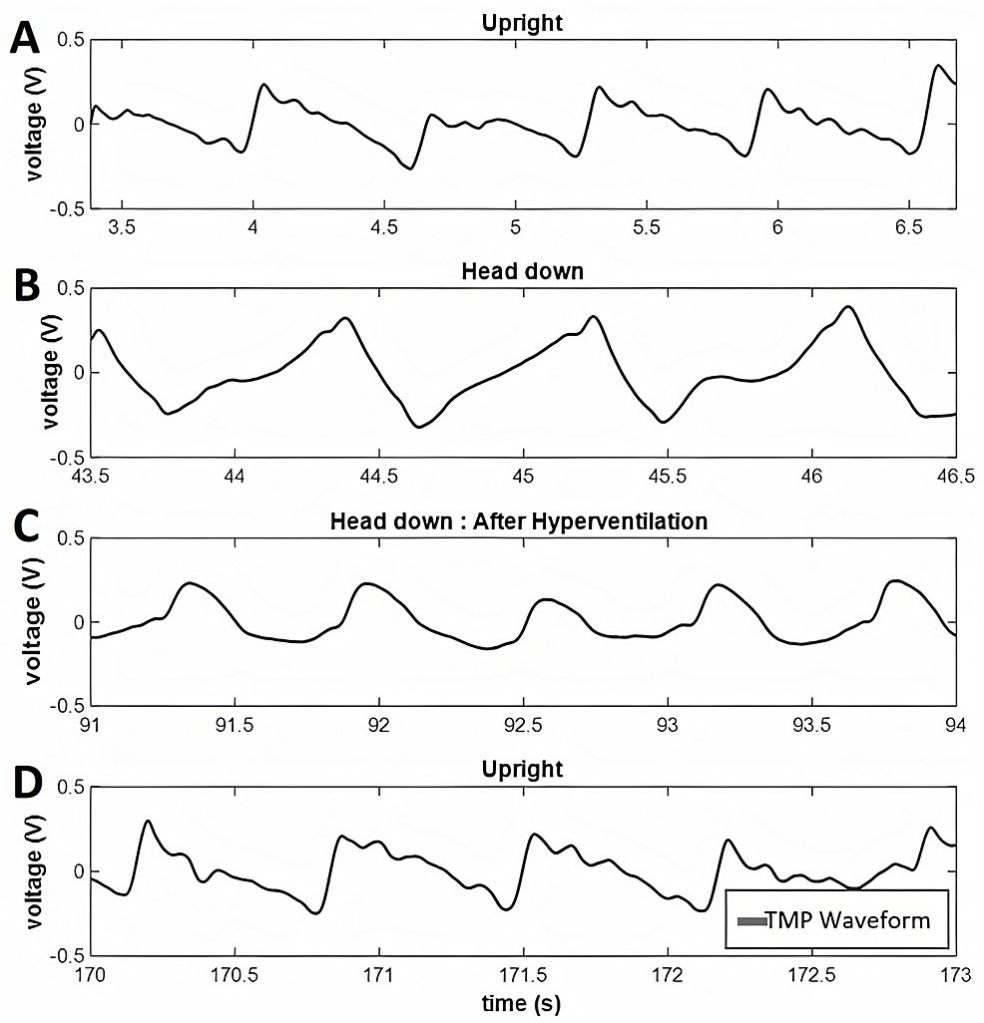
Example tympanic membrane pulsation waveforms This figure shows an example trace from tympanic membrane pulsation recording of a single subject during (A) resting baseline (upright), (B) tilt-testing, (C) hyperventilation, and (D) recovery. TMP: tympanic membrane pulsation

**Figure 5:**
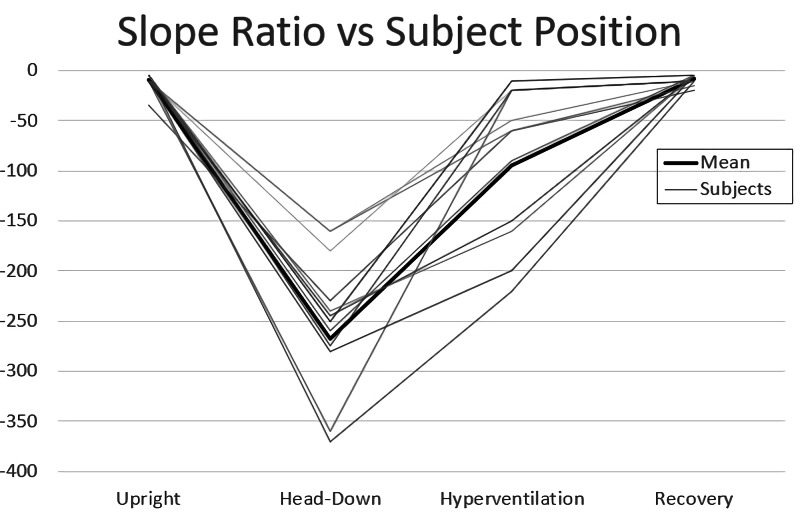
Plot of slope ratio versus provocative tests The plot of slope ratio versus provocative maneuver across the 14 subjects in the second cohort.

Second, “can alterations in ICP be detected by the brain stethoscope?” An example of measured TM pulsations at rest can be seen in Figure 4A. When the subject’s ICP is elevated during the head down a portion of the tilt-testing, the rise time of the wave is delayed, and the peak is shifted to the right (Figure 4B). When the ICP decreases back towards the baseline during hyperventilation, the rise time decreases closer to the original upright resting waveform (Figure 4C). This is followed by a complete return to a normal waveform after the short upright resting period (Figure 4D). This trend persists across all subjects as seen in Figure 5 where the SR for each study subject is plotted for all subjects during each maneuver.

Third, “do we see the expected physiological trends?” In Figure 4, a decrease in SR (delay in waveform rise time) is when elevated ICP is induced by tilt testing. This is followed by an increase in SR when ICP is then decreased with hyperventilation and a complete return to baseline with the post-experimental recording. This trend is expected for TMP associated with ICP. The results of paired t-testing of the subjects’ SR value during each provocative maneuver are summarized in Table 1. A statistically significant difference in TMP waveform morphology can be seen between the upright resting and tilt-testing maneuvers (elevated ICP) as well as between the tilt-testing and hyperventilation (reduced ICP) phases. There are no statistically significant differences between the pre-test and post-test baseline recordings. Thus, in this pilot study, there are detectable reversible alterations in TMP morphology in a model of induced ICP elevations using the brain stethoscope approach. 

**Table 1:** Paired t-test analysis of slope ratios during provocative maneuvers Paired t-test analysis comparing the slope ratios between the provocative maneuvers. This demonstrates a statistically significant change in subjects’ tympanic membrane pulsation waveform with alterations in intracranial pressure status.

Paired t-test	
Challenge	p-Value
Upright versus head down	1.3x10^-9^
Head down versus hyperventilation	1.9x10^-7^
Upright versus recovered	0.43

Finally, “can SR be used to predict ICP status?” An SR of -51 was determined to be three standard deviations from the mean SR of the baseline recordings from the first cohort. This number was used as the threshold for normal ICP for classifying all future recordings. Using his threshold, the algorithm was able to correctly classify 18 of 18 recordings of the first cohort as either normal or elevated ICP. When the classification algorithm was applied to the second cohort it was able to correctly classify all 42 new recordings as either normal or elevated ICP with 100% sensitivity and specificity (Table 2).

**Table 2:** Results of classification algorithm This table shows the results of the classification algorithm on the trained and untrained data including the sensitivity and specificity of the algorithm. ICP: intracranial pressure

	True + (Raised ICP)	True - (Normal ICP)	False +	False -
Untrained data (n=18)	6	12	0	0
Trained data (n=42)	14	28	0	0
Total	20	40	0	0
Sensitivity	100%	Specificity	100%	

## Discussion

The slope ratio method of waveform analysis showed consistent and stable changes with increased ICP and abnormal brain compliance across 20 trials in 17 healthy volunteers. Analysis of individual TM readings were correctly classified as normal or elevated ICP in 60 of 60 measurements using a threshold-based algorithm. This suggests that the “brain stethoscope” is capable of reliably differentiating ICP changes in healthy subjects, which would allow the prediction of patients’ ICP status. Previous attempts to utilize the cochlear aqueduct to identify raised ICP have failed due, at least in part, to the unreliability of classical waveform metrics (amplitude/time average mean) and the requirement of bulky equipment. Utilizing the “brain stethoscope” with the slope ratio method offers an alternative to previous methods and metrics. The SR metric was found capable of consistently identifying elevated ICP in this pilot study. This represents a significant step towards developing a non-invasive screening tool for identifying subjects with raised ICP. Several approaches for noninvasive ICP monitoring have been proposed, each with strengths, but also limitations. These include optic nerve sheath width, optical coherence tomography for macular depth, transcranial ultrasound transmission time, transcranial high-frequency radiofrequency impedance, and transocular ultrasound among others [2,5,6]. The strengths of the brain stethoscope include the derivation of a real-time waveform, easy deployment among hospital, clinic, and home environment, and straightforward adaptability to telemedicine for the conditions where longitudinal measurement of ICP and brain compliance may inform better management, specifically shunted hydrocephalus and idiopathic intracranial hypertension. In this last group, the patient's baseline measurements become his own control. The technique also involves no complicated or bulky equipment and is inexpensive to fabricate. However, more work is required before this approach is ready to be used in clinical practice as this study has several major limitations.

There are four limitations to this study and approach. First, the study was performed on healthy individuals, not patients with pathologically elevated ICP. While the proposed approach demonstrated a high potential for altered ICP detection, we have not yet proven its efficacy in patients with pathological ICP. Second, the number of subjects is small and the study needs to be repeated in prospective larger trials on actual patients with pathologically elevated ICP such as pseudotumor cerebri, hydrocephalus, or occluding mass. Third, ICP was not directly measured in the subjects, but alterations in ICP were presumed to occur with physiological maneuvers that were previously documented in the literature. Hence, we have demonstrated a qualitative association. However, future studies with direct ICP measurements will be needed to validate the proposed method. Fourth, this approach requires subjects to have intact outer, middle, and inner ear anatomy and would likely not function in subjects with bilateral ear pathology such as ruptured tympanic membranes, otosclerosis, or major ear trauma.

## Conclusions

In summary, this paper has four conclusions. First, that the "brain stethoscope" can detect increased ICP from the TM pulsation waveform in healthy subjects. Second, analysis of the TM waveform using slope ratio calculations is capable of distinguishing normal versus induced elevated ICP. Third, the tilt and hyperventilation maneuvers showed the expected physiologic trends. Lastly, further studies are needed on patients with pathological ICP. If the current results can be duplicated in patients with pathologically elevated ICP, then the "brain stethoscope" may prove to be a practical, inexpensive tool to objectify and facilitate monitoring and diagnosis of elevated ICP. Therefore this technique may prove to be of use in certain clinical conditions where abnormal intracranial pressure is a key therapeutic triage or management concern, including hydrocephalus and shunt malfunction, idiopathic intracranial hypertension, head trauma, and stroke.
